# Ontogeny of Tissue-Resident Macrophages

**DOI:** 10.3389/fimmu.2015.00486

**Published:** 2015-09-22

**Authors:** Guillaume Hoeffel, Florent Ginhoux

**Affiliations:** ^1^Singapore Immunology Network (SIgN), Agency for Science, Technology and Research (A*STAR), Singapore, Singapore

**Keywords:** macrophages, monocytes, fetal liver, yolk sac, C-Myb, erythro-myeloid progenitors, hematopoiesis, hematopoietic stem cells

## Abstract

The origin of tissue-resident macrophages, crucial for homeostasis and immunity, has remained controversial until recently. Originally described as part of the mononuclear phagocyte system, macrophages were long thought to derive solely from adult blood circulating monocytes. However, accumulating evidence now shows that certain macrophage populations are in fact independent from monocyte and even from adult bone marrow hematopoiesis. These tissue-resident macrophages derive from sequential seeding of tissues by two precursors during embryonic development. Primitive macrophages generated in the yolk sac (YS) from early erythro-myeloid progenitors (EMPs), independently of the transcription factor c-Myb and bypassing monocytic intermediates, first give rise to microglia. Later, fetal monocytes, generated from c-Myb^+^ EMPs that initially seed the fetal liver (FL), then give rise to the majority of other adult macrophages. Thus, hematopoietic stem cell-independent embryonic precursors transiently present in the YS and the FL give rise to long-lasting self-renewing macrophage populations.

## Introduction

Ilya (Elie) Metchnikoff first described the mechanism of phagocytosis and the cells responsible for this process over a century ago. These professional phagocytic cells were named “macrophages” (from the Greek derivation macro = large and phage = devouring, “large devouring cells”). These were separate from “microphages,” which included polymorphonuclear phagocytes (1). Determining the role of macrophages in pathogenic infections was one of the fundamental observations leading to the concept of cellular immunity (2). Through this seminal work, Metchnikoff anticipated the central role of macrophages in tissue inflammation and homeostasis. We recommend an elegant historical review for more details about Metchnikoff’s work by Yona and Gordon in this issue (3).

Since then, the definition of the phagocyte system has been continuously refined, and our understanding of the wide-ranging functions of macrophages has been substantially expanded. It is now clear that, in addition to their classical function in the activation and resolution of tissue inflammation, macrophages also play roles in tissue-specific functions, tissue remodeling during angiogenesis and organogenesis, and wound healing, to name a few (4). Macrophages are exquisitely adapted to their local environment, acquiring organ-specific functionalities during developmental stages and the steady state (4). Macrophages are able to support multiple tissue functions, integrating cues from both the outside environment and their microenvironment to act as rheostatic cells of tissue function. Thus, tissue-resident macrophages represent an attractive target for modern medicine to treat a wide spectrum of diseases in which they have been implicated, including atherosclerosis, autoimmune diseases, neurodegenerative and metabolic disorders, and tumor growth (5–8). Understanding the origin and developmental pathways of macrophages will help to design novel intervention strategies targeting these cells in tissue-specific sites.

A number of observations now indicate that certain macrophage populations derive from embryonic precursors sequentially seeding tissues during development (9–13). Two macrophage progenitors, yolk sac (YS) macrophages and fetal monocytes, have been described in the embryo, but their exact nature and origin were not fully understood until recently (14, 15). Here, we discuss recent developments in our understanding of the origin of adult tissue-resident macrophages, exploring the sequence of progenitors generated during embryonic and adult hematopoiesis. We focus on the relative contributions of YS macrophages and fetal or adult monocytes, including a discussion of our own recent data exploring the heterogeneity of fetal monocyte developmental pathways.

## Early Concepts

Macrophages form part of the mononuclear phagocyte system (MPS), which also includes circulating monocytes and dendritic cells (16). Until recently, our vision of macrophage origin and homeostasis was largely based on seminal studies that used *in vivo* radioisotope labeling and radiation chimera experiments. These studies led to the early dogma that resident macrophages were constantly replenished from circulating bone marrow (BM)-derived monocytes as a continuum of differentiation (17–19).

In agreement with that concept, studying the ontogeny of the MPS revealed that monocytes and macrophages derived from macrophage and dendritic cell progenitors (MDPs) present in the BM, which are phenotypically defined as lineage-c-kit^+^CX3CR1^+^Flt3^+^CD115^+^ (20). MDPs further differentiate through a newly described common monocyte precursor (cMoP), phenotypically defined as lineage^−^c-kit^+^CX3CR1^+^Flt3^−^CD115^+^ (21), that gives rise to the two main subsets of circulating monocytes distinguished by the expression of Ly6C (22).

Specific tissue macrophages, such as dermal, gut, and heart macrophages, seemed to follow the model of Van Furth, that macrophages are derived from monocytes (23–25). However, this model did not fit in all cases and evidence also emerged indicating that macrophages were long-lived cells, able to self-renew locally. Hashimoto was the first to speculate that Langerhans cells (LCs) represented a self-perpetuating “intraepithelial phagocytic system” (26). Performing a human skin transplantation assay onto nude mice, Krueger et al. described the remarkable longevity of LCs, which were able to persist in the grafts for more than 2 months (27). Their ability to self-renew through proliferation was later described using DNA densitometry (28). Similar conclusions were drawn soon after regarding alveolar macrophages (29). The dominant concept of “the monocytic origin” of tissue macrophages was also challenged through experiments in animals with prolonged monocytopenia following strontium-89 monocyte depletion, in which liver Kupffer cells were shown to maintain cell numbers by increasing local proliferation (30, 31).

More recently, the use of long-term parabiotic mice and subsequent fate-mapping models have challenged the MPS paradigm and revealed that, unlike all other hematopoietic cells, which rely on hematopoietic stem cell (HSC)-derived BM progenitors, certain macrophage populations possess the unique ability to self-renew locally independently of circulating precursors (32–36). Initial studies describing the presence of macrophages in embryonic tissues suggested that tissue macrophages derived from embryonic progenitors. In rodents, macrophage-like cells first described in the brain rudiment and in the developing skin (37, 38) were named “fetal macrophages” and found to exhibit a high capacity for proliferation (39). These observations suggested that adult macrophages derive from fetal macrophages established during early development. However, whether these fetal macrophages were maintained until adulthood or were replaced postnatally was not addressed until recently. In addition, the exact nature and the origin of fetal macrophage progenitors remained unclear.

## Embryonic Hematopoiesis

**Figure 1:**
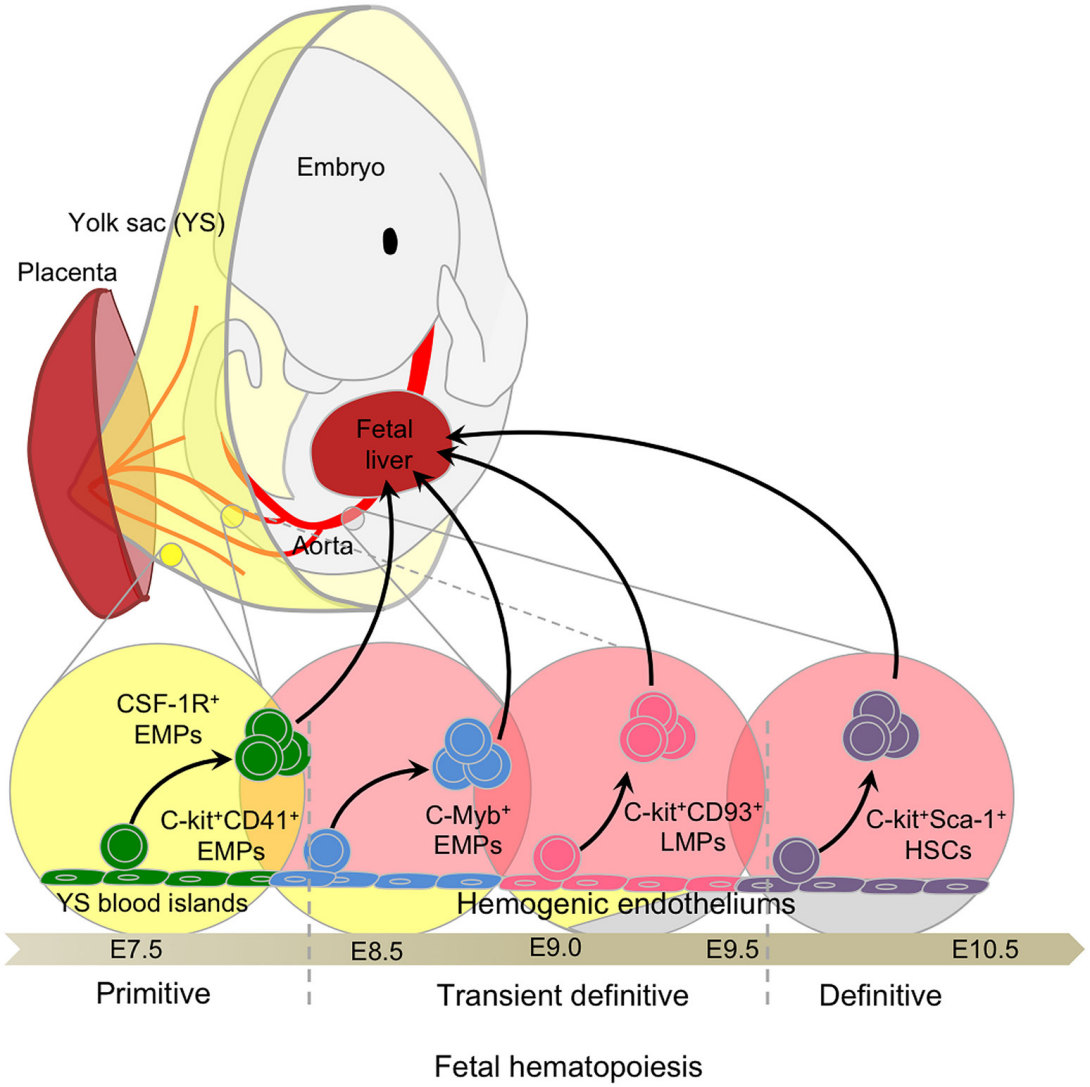
**Fetal hematopoiesis**. Primitive, transient definitive, and definitive waves of fetal hematopoiesis sequentially generate progenitors able to seed the fetal liver. Primitive hematopoiesis starts at E7.0 in the blood islands of the extra-embryonic yolk sac (YS) and generates erythro-myeloid progenitors (EMPs). Early EMPs initially express CD41 and later, CSF-1R, a signature of myeloid/macrophage commitment. Concomitant to the establishment of the blood circulation at E8.5, the YS hemogenic endothelium (HE) generates late EMPs expressing C-Myb. At approximately E9.0, the intra-embryonic mesoderm generates additional HE and emerging progenitors with lymphoid potentials (LMPs) without long-term reconstitution (LTR) capacity. These C-Myb^+^ EMPs and LMPs constitute the so-called transient definitive wave. Finally, hematopoietic stem cells (HSCs) with LTR activity emerge from the main HE situated in the aorta-gonad-mesonephros (AGM) regions and in the placenta.

Mammalian embryos produce several transient waves of hematopoietic cells before the establishment of HSCs in the BM during late gestation (40, 41). The multiple embryonic waves are differentially regulated in time and space and exhibit distinct lineage potentials. Importantly, they contribute to hematopoietic populations that persist until adulthood. These waves include primitive hematopoiesis in the YS, and definitive hematopoiesis, which comprises a transient definitive stage, generating multi-lineage erythro-myeloid progenitors (EMPs) and lympho-myeloid progenitors (LMPs), and a definitive stage characterized by the production of HSCs in the aorta-gonad-mesonephros (AGM). These transient progenitors establish themselves transiently in the fetal liver (FL) during the mid to late stages of hematopoiesis. The sequential waves of hematopoiesis can overlap in time and space (Figure 1) and remain difficult to separate clearly, even with the most recent fate-mapping tools available.

### Primitive hematopoiesis

**Figure 2:**
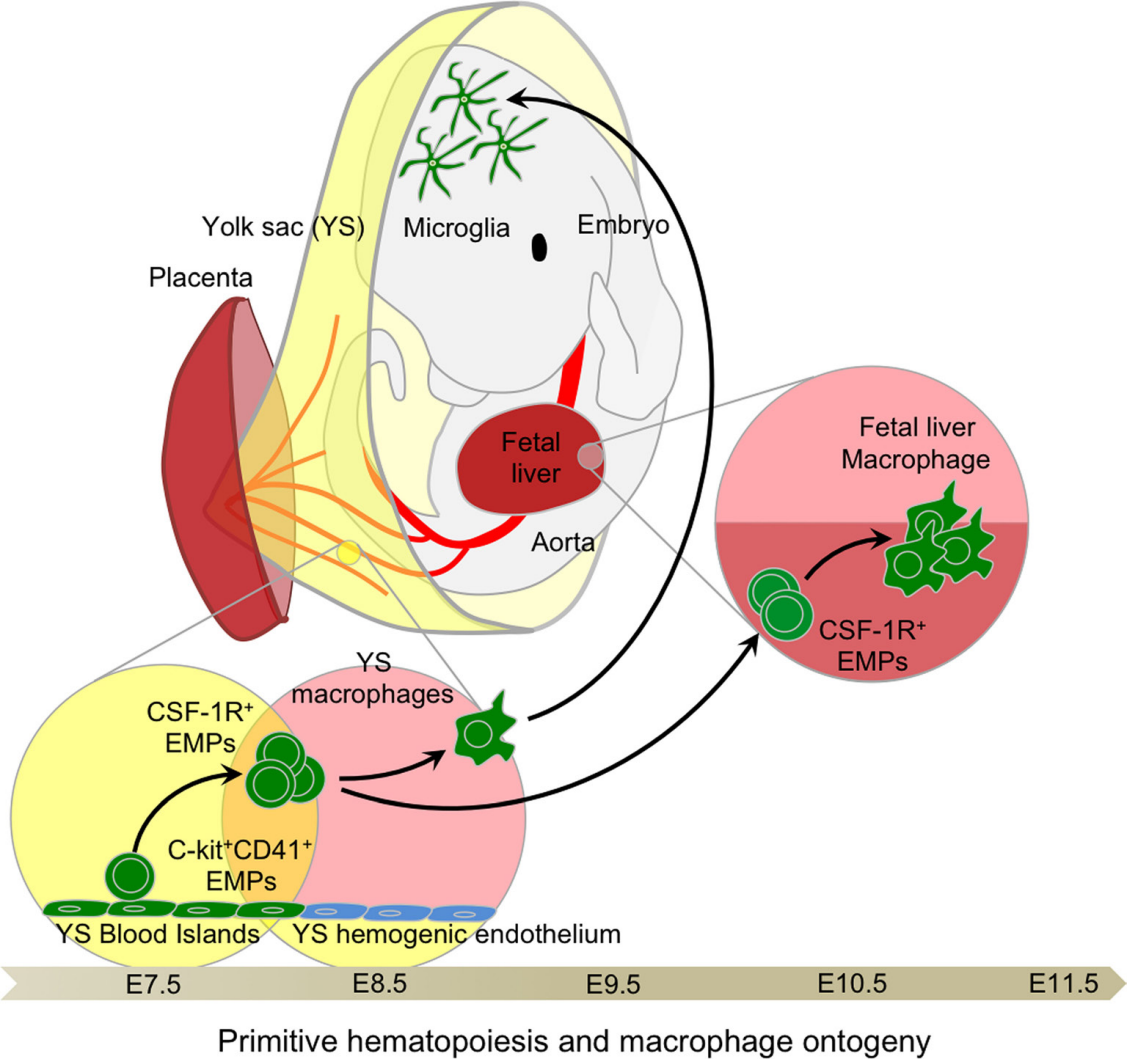
**Primitive hematopoiesis and yolk sac macrophage ontogeny**. Early EMPs emerge in the YS around E7.5 before establishment of the blood circulation. They express CD41 and CSF-1R and are independent of the transcription factor C-Myb. Upon establishment of the blood circulation around E8.5, EMPs differentiate into primitive macrophages as well as primitive erythrocytes and granulocytes. Primitive macrophages seed all fetal tissues, in particular the head where they will give rise to future brain microglia that are able to continuously self-renew throughout adulthood. EMPs seeding the fetal liver briefly expand to generate a local macrophage population, likely important for sustaining enucleation of primitive erythrocytes passing through the sinusoid prior to the establishment of definitive hematopoiesis and the generation of fetal monocyte-derived macrophages in the fetal liver.

In mice, the first hematopoietic progenitors appear in the extra-embryonic YS blood islands at around embryonic age 7.25 (E7.25), where primitive hematopoiesis is initiated, producing mainly nucleated erythrocytes. This observation linked the myeloid progenitors observed in the YS at E7 with the emergence of YS macrophages after E9.0 [Figure 2; Ref. (42–45)]. Primitive hematopoiesis was also shown to produce megakaryocyte progenitors (46). The denomination “primitive” was given to reflect the production of embryonic erythroblasts, like those observed in lower species such as fish, amphibians, and birds, and remaining nucleated throughout their life span (47–49). This denomination was extended to macrophages in the YS due to their concomitant development prior to FL hematopoiesis. Interestingly, no clear evidence of monocytic intermediates was reported at this stage, although the seminal study of Cline and Moore did mention the existence of local intermediate cells between progenitors and functional macrophages (43). Studies by Naito and Takahashi et al. clarified the emergence of primitive macrophages in the YS blood islands in the mouse and rat, observing an absence of endogenous peroxidase activity as a surrogate marker for an absence of monocytic intermediates, such as those found in the BM (50–52), suggesting a unique developmental pathway for YS macrophages (53, 54).

### Transient definitive hematopoiesis

**Figure 3:**
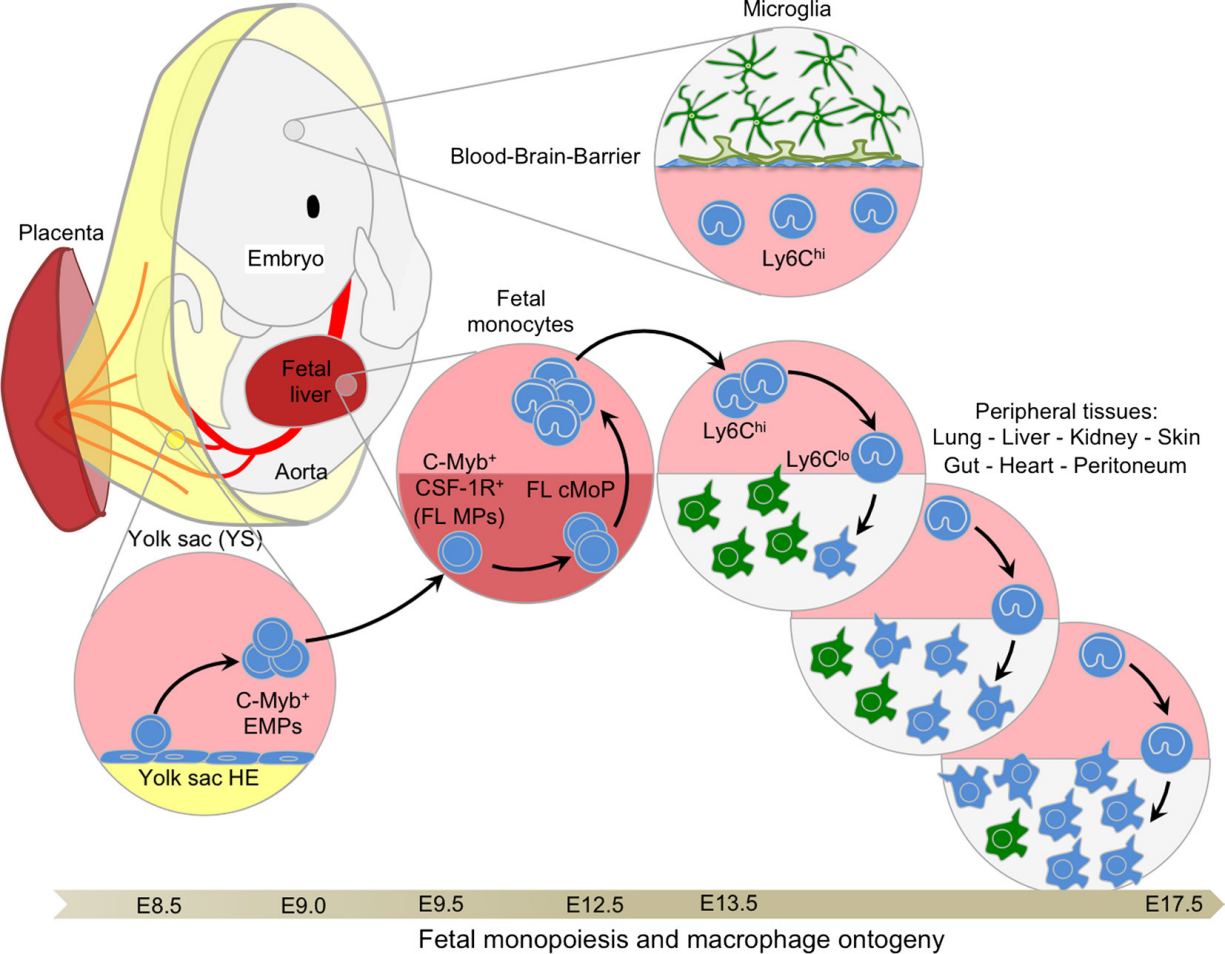
**Fetal monopoiesis and macrophage ontogeny**. At E8.5 as the blood circulation is established, the YS HE, possibly in conjunction with other hemogenic sites, generates EMPs expressing CD41 and c-Myb. These late EMPs seed the fetal liver around E9.5 where they expand rapidly to give rise to CSF-1R^+^ myeloid progenitors that are able to generate fetal monocytes through cMoP intermediates at E12.5. Fetal monocytes then spread via the blood circulation to all tissues, with the exception of the brain, which is isolated by the establishment of the blood–brain barrier at approximately E13.5. In the tissues, fetal monocytes begin to differentiate into macrophages, progressively outnumbering the previously established primitive macrophages. These fetal monocyte-derived macrophages maintain the capacity to self-renew throughout adulthood in certain tissues, such as the liver or the lung, where they will not be replaced by adult BM-derived monocytes.

The quest to elucidate the origins of embryonic HSCs led to the discovery of earlier lineage-restricted HSC-independent progenitors seeding the FL at E10.5. These progenitors arise concurrently with the transition of primitive to definitive erythropoiesis and were thus considered to form a transient stage of definitive hematopoiesis (45, 47, 55). Transient definitive hematopoiesis consists of progenitors sequentially acquiring myeloid, then lymphoid potential, without exhibiting the long-term reconstitution potential of HSCs. Seminal work from Palis and colleagues on embryonic erythropoiesis in the YS described the parallel emergence of multiple myeloid lineage potential progenitors from E8.25 in the YS (45). Palis et al. first observed the emergence of definitive progenitors for mast cells and a bipotential granulocyte/macrophage progenitor. These progenitors then migrated to the FL through the bloodstream after E8.5, once circulation was established (44, 56). From this pattern of development, the authors concluded that definitive hematopoietic progenitors arise in the YS, migrate through the bloodstream, and seed the FL to rapidly initiate the first phase of intra-embryonic hematopoiesis. Similarly, primitive and definitive erythropoiesis, associated with myelopoiesis, was also shown to emerge prior to HSC in the zebrafish embryo (57). Bertrand et al. showed that definitive hematopoiesis initiates in the posterior blood island with only transient proliferative potential. Because these HSC-independent definitive progenitors were observed to produce definitive erythroid and myeloid cell types, but not to colonize the zebrafish thymus (implying that they are devoid of lymphoid potential), this population was termed EMPs (57). Interestingly, EMPs can also emerge from the hemogenic endothelium (HE) located in the placenta and umbilical cord (58) and colonize the FL from E9.5 (55) to participate in definitive hematopoiesis. Further studies advanced the field significantly by identifying CD41 as an early marker (pre-CD45) for defining hematopoietic progenitors, including EMPs, emerging from the YS (59, 60). Altogether, these important studies provided phenotypic and functional analyses of the first hematopoietic progenitors and demonstrated that definitive hematopoiesis proceeds through two distinct waves during embryonic development (Figure 3).

In parallel, several groups have also identified other multipotential progenitors with lymphoid- or myelo-lymphoid-restricted potential in the YS and the developing para-aortic splanchnopleura (P-Sp) prior to HSCs (61, 62). Lacaud et al. also described AA4.1 (CD93)^+^ multipotential progenitors present in the E14.5 FL with T cell, B cell, and macrophage potential (63), although the precise origin of these progenitors was not addressed. At the same time, the team of Jacobssen identified Flt3^+^ lympho-myeloid progenitors (LMPs) devoid of erythrocyte and megakaryocyte capacity (64). Later, cells with myelo-erythroid and lymphoid lineage potential, such as B-1 cells present in the adult spleen, were associated with E9.5 YS progenitors expressing AA4.1 and CD19 (65). A year later, the same team also identified T cell potential within the E9.5 YS progenitors (66). Using a Rag-1-Cre fate-mapping model, Boiers et al. confirmed that these LMPs emerged at approximately E9.5 in the YS, seeding the FL by E11.5 to give rise to T and B cells, as well as granulocytes and monocytes in the E14.5 FL, prior to HSCs (67). Finally, lympho-myeloid progenitors isolated from the dorsal aorta at E9.0 were shown to acquire long-term reconstitution capacity after a few days of *in vitro* culture with stromal cells, and were called immature HSCs (68). Without preculture, these multipotential progenitors can only engraft natural killer (NK)-deficient Rag2γc^−/−^ mice. Whether these immature HSCs arise from LMPs or represent a distinct wave of progenitors remains to be clarified. However, these seminal studies provided strong evidence that lymphoid potential can emerge from the YS, prior to HSC-budding from the AGM (69).

Because the emergence of EMPs and LMPs overlaps in time and space, they could not be distinguished clearly until recently. Previous reports had suggested that lymphoid potential was restricted to the CD41-negative cell fraction (59, 65). However, CD41 is also expressed in a sub-fraction of FL HSCs, and so this phenotypical distinction spread some confusion (47). Finally, a recent report from the group of Palis clarified this point by showing that co-expression of c-kit, CD41, and CD16/32 defines EMPs and allows their separation from other progenitors with lymphoid potential, such as those giving rise to the B-1 cell (70). McGrath et al. extended the notion of EMPs by showing their potential to generate neutrophils, megakaryocytes, macrophages, and erythrocytes. Finally, transplantation of EMPs in immune-compromised adult mice can also provide transient adult red blood cell reconstitution (70).

To conclude, commitment to hematopoietic fates begins during gastrulation in the YS, which represents the only site of primitive erythropoiesis and also serves as the first source of transient definitive hematopoietic progenitors. HE develops from the YS to various intra-embryonic sites, and acquires myeloid and then lymphoid lineage potentials in overlapping waves, highlighting the complexity of the hematopoietic output. Whether some of these progenitors arise from independent sources or represent different maturation stages of a shared hematopoietic wave, culminating with the generation of HSCs, needs to be further clarified. However, it is tempting to speculate that the clear contrasts in differentiation/lineage potential do not reside in their intrinsic potential, but rather in the extrinsic signals provided by the local environment.

### Definitive hematopoiesis

**Figure 4:**
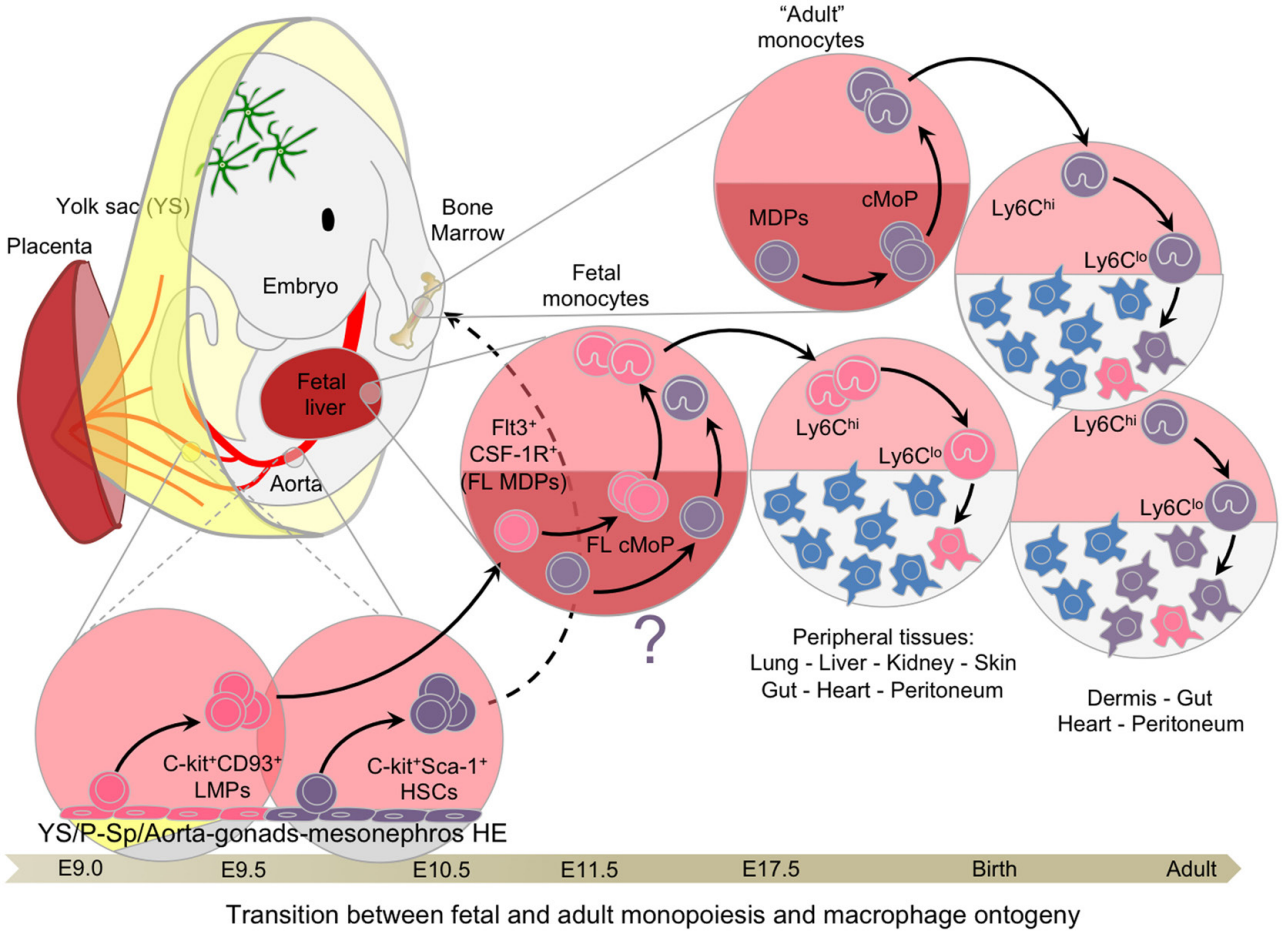
**Transition between fetal and adult hematopoiesis**. Hemogenic endothelial cells from extra and intra-embryonic hematopoietic tissues generate C-Myb-dependent multipotential progenitors, such as LMPs and pre-HSCs, between E9.0 and E10.5, culminating with the emergence of mature HSCs with long-term reconstitution-bearing potential. CD93 (AA4.1) expression is associated with the emergence of lymphoid potential, whereas Sca-1 is the hallmark of HSCs. These progenitors seed the fetal liver around E10/E11, expanding and giving rise to the various lineages of the hematopoietic system, including fetal monocytes. These late fetal monocytes continue to participate in the tissue-resident macrophage network until hematopoiesis switches completely from the fetal liver to the bone marrow. Once adult hematopoiesis begins to take place in the bone marrow generating monocytes, certain tissues, such as the dermis, heart peritoneum, and the gut, continue to recruit adult monocytes to generate resident macrophages and replace with time the embryonic-derived macrophages.

The complex hierarchy of stem and progenitor cells in the BM is first established during embryonic development starting with the emergence of small numbers of HSCs from the AGM at E10.5 in murine embryos or at 5 weeks in human embryos (71, 72). After E9.5 in the mouse, with the determination of the intra-embryonic mesoderm toward a hematopoietic lineage, new waves of hematopoietic progenitors emerge within the HE of the embryo proper (Figure 4), first in the P-Sp region and the umbilical and vitelline arterial regions of the embryo, then in the AGM region and the placenta (55, 73, 74). The hematopoietic activities of the P-Sp and AGM first generate immature HSCs and then mature HSCs, which are defined by their capacity to reconstitute adult conventional mice (long-term reconstitution; LTR). Both immature and mature HSCs seed the FL at approximately E10.5 (68, 71, 75, 76) to establish definitive hematopoiesis (40, 77, 78). A maturation step seems necessary for immature HSCs to express their LTR activity in full, which is then maintained until adulthood (68). However, further investigations using a fate-mapping system would be necessary to confirm this model.

The FL becomes the major hematopoietic organ after E11.5, generating all hematopoietic lineages. Importantly, the FL itself does not produce progenitors *de novo*, but rather recruits progenitors derived from the YS and other hemogenic sites, to initiate definitive hematopoiesis (79) in parallel with the expansion of the definitive HSC population before their migration to the spleen and BM (80).

**Figure 5:**
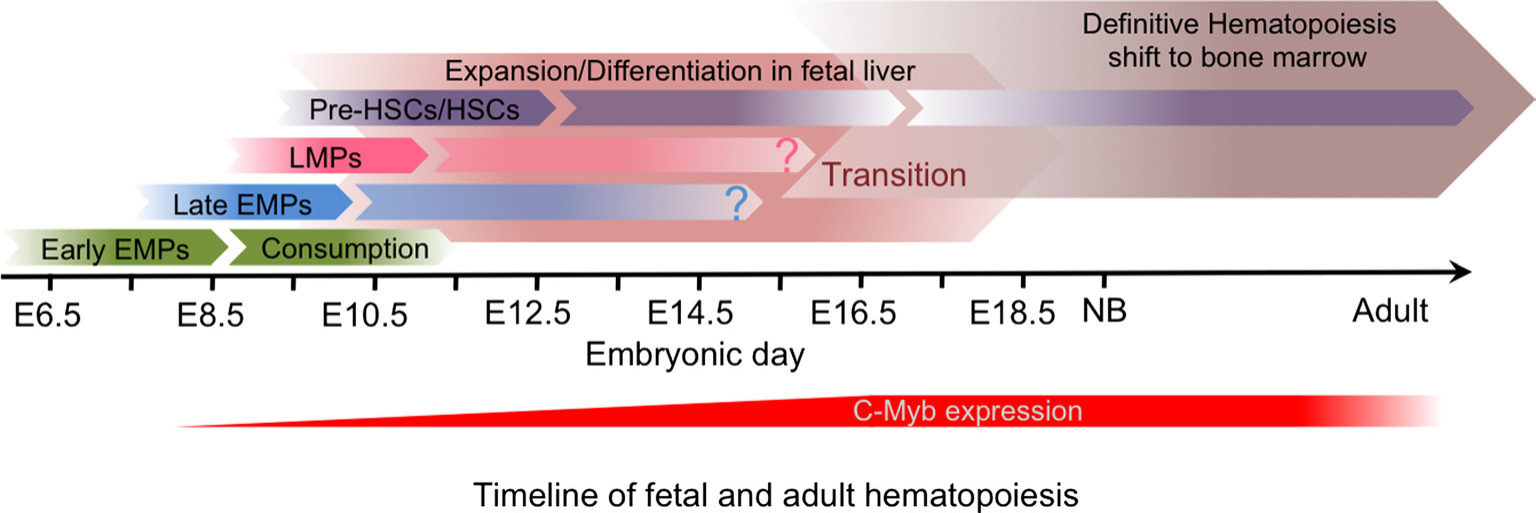
**Timeline of fetal and adult hematopoiesis**. The primitive hematopoiesis is initiated in the yolk sac independently of C-Myb activity, and generates early CSF-1R^+^ EMPs that give rise to YS macrophages without monocytic intermediates during a short time window and will establish the brain microglia. The transient definitive hematopoiesis and then the definitive hematopoiesis are both dependent on C-Myb activity and generate progenitors that differentiate in the fetal liver. The transient definitive wave, which include EMPs and then LMPs, give rise in particular to fetal monocytes that seed the tissues prior to birth to establish the self-renewing tissue-resident macrophage network. Although only HSCs, which result from the definitive hematopoiesis, seem to be maintained in the bone marrow in adults, the relative contribution of the transient definitive wave to the adult immune system remains unclear.

**Figure 6:**
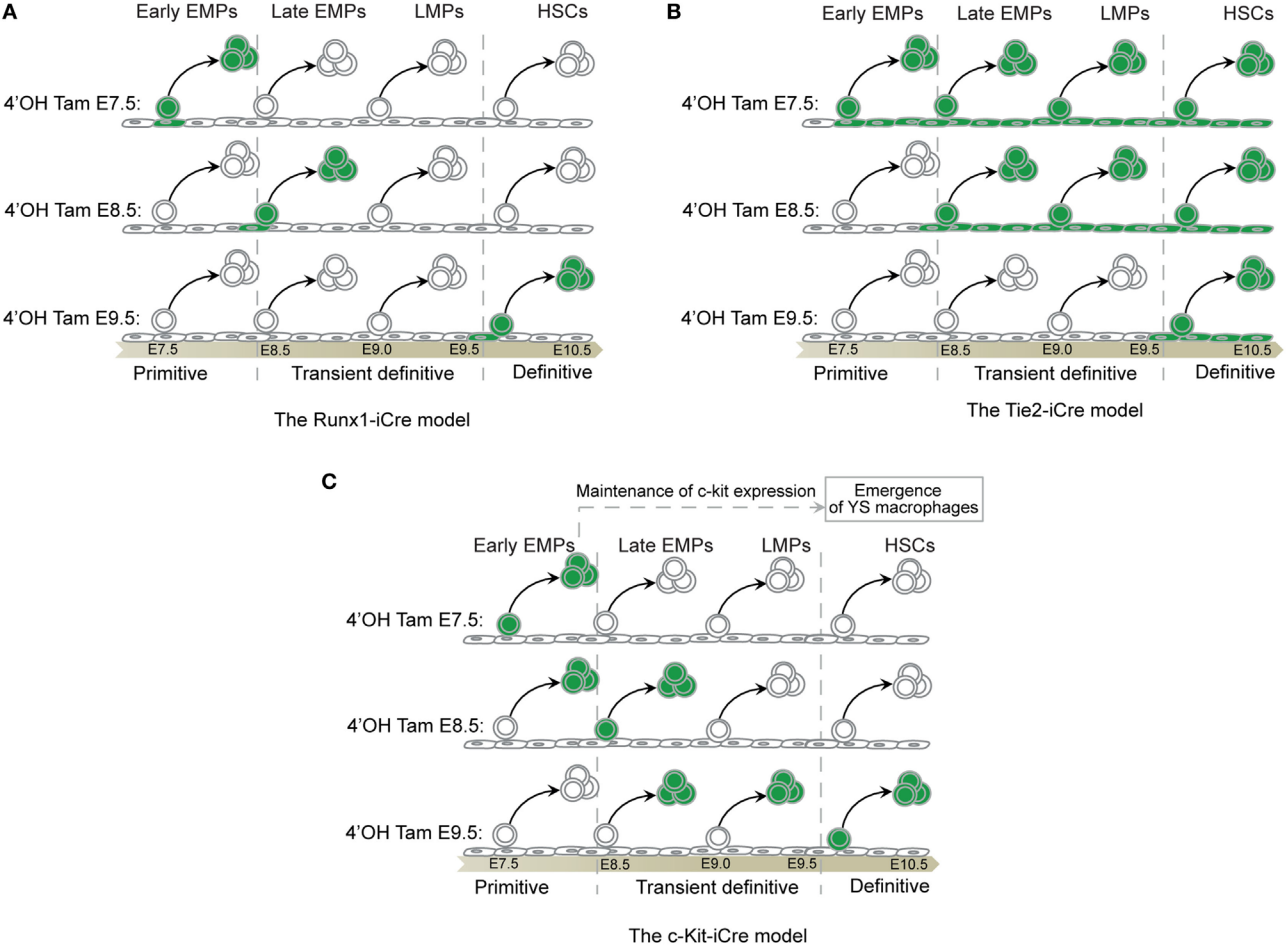
**Fate-mapping systems**. **(A)** The Runx-iCre fate-mapping model (86) used in our study targets the hemogenic transition (88), hence, labeling specifically progenitors in the process of budding out from the hemogenic endothelium. The Runx1 expression decreases in progenitors once they start to express Vav thus reducing the chances of tagging released progenitors from precedent waves (88). As a consequence, Runx-iCre tagging is restricted to a short time window in the lifespan of a given progenitor and allows a sharp definition of each hematopoietic wave. However, this model also restricts the tagging to only a small fraction of the targeted progenitor wave. **(B)** Tie2 is expressed in all endothelial cells that constitute the hemogenic endothelia even before the hemogenic transition (89). Thus, all endothelial cells and their progeny (non-hematopoietic and hematopoietic cells) will be labeled after tamoxifen injection using the Tie2-iCre model. As a consequence, an early tamoxifen injection (such as at E7.5) will result in the tagging of all hematopoietic cells emerging before the time of analysis. This will include progenitors from the primitive, the transient definitive, and the definitive waves if, for example, the analysis is done at E11.5. A late injection (such as at E10.5) will restrict the tagging to only the latest hematopoietic stem cells wave as they are just budding from HEs (90). Thus, this model might not be suitable to clearly separate the primitive from the transient definitive waves of hematopoiesis. However, this model could be important to study late HSC progeny as no other progenitors than HSCs emerge from HE after E10.5 (91). **(C)** C-kit is expressed by all hematopoietic progenitors and does not label endothelial cells that constitute the HEs (89). An early tamoxifen injection (such as at E7.5) will restrict the labeling to early progenitors making suitable the c-kit-iCre model to study the primitive hematopoiesis. However, the FL recruits progenitors of each hematopoietic wave from E8.5 until E11 (79). These progenitors still express c-kit and coexist after seeding the FL during the time necessary for their differentiation (47, 55). A later tamoxifen injection (such as at E9.5) might thus result in the cumulative labeling of undifferentiated primitive and definitive progenitors, including the transient wave of EMPs and LMPs. Thus, such model may not be suitable to resolve the complexity of the different embryonic hematopoietic waves characterized by short time windows of emergence and strong overlapping tendencies. Primitive hematopoietic progenitors are rapidly consumed and the engagement of EMPs and LMPs in FL hematopoiesis reduces the expression of c-kit on their surface. Thus, later tamoxifen injection (such as at E11.5) could restrict the labeling to newly derived HSCs expressing high level of c-kit without labeling precedent progenitor waves (87). Such model might be interesting to study the progeny of late HSCs although the risk of tagging the progeny of EMPs and LMPs or later committed progenitors derived from HSCs remains high and difficult to exclude. Further analysis would be necessary to clarify the potential of such model.

The contribution of HSCs to FL hematopoiesis is complex to evaluate, partly because of the lack of specific fate-mapping models, and also the relatively limited knowledge regarding embryonic HSC maintenance and homeostasis in this environment. The capacity for long-term reconstitution, which defines functional HSCs, is present in the AGM by E10.5 (76). However, lineage-specific commitment may not occur *in vivo* immediately after reaching the FL environment. A number of other progenitors generated during transient definitive hematopoiesis, as discussed above, are already present and able to give rise to almost all cell lineages, which could prevent HSC consumption and differentiation (Figure 5). Evaluation of HSC contribution has long been based on the assumption that all hematopoietic cells in the FL were derived from HSCs as is the case in the BM (81). Many multipotential progenitors share the same phenotype with pre-HSC and HSCs, such as the expression of CD41 and AA4.1 (60), adding to this confusion. The combination of the marker Sca-1 and new markers such as those from the SLAM family (82) have greatly helped to clarify the characterization of HSCs, defined now as Lin^−^ckit^+^Sca-1^+^CD150^+^CD48^−^CD244^−^. However, no specific fate-mapping model exists to characterize embryonic HSC progeny with the exception of the Flt3-Cre model (83), which was used until now with the assumption that embryonic and adult HSCs follow similar differentiation pathways. Our recent report suggests that the Flt3-Cre model can also be used to follow the progeny of LMPs (15). Furthermore, in the nascent BM, the long-term repopulation (LTR) capacity that characterizes functional HSCs is only observed at around E17.5 (84). Considering the time required to initiate full HSC differentiation, these data suggest that proper adult HSC-derived hematopoiesis does not take place in the BM until a few days after birth. Characterization of the functional specificities and regulatory pathways of HE that give rise to HSCs versus those that generate EMPs and other multipotential progenitors could aid the development of new fate-mapping models and improve our understanding of this process (85). Use of other fate-mapping models such as the Runx1-Mer-Cre-Mer (Runx1-iCre) (86), Tie2-Mer-Cre-Mer mice (14), and the c-kit-Mer-Cre-Mer mice (87) provided complementary results, although a careful analysis of the targeted cells in time and space is not yet fully available for the last two models. We present here our best interpretation of the data provided in these two recent studies that have used these models in light of the literature and our own results and experience using the Runx1-iCre model (Figure 6).

## Embryonic and Adult Precursors of Adult Tissue-Resident Macrophages

### Yolk sac macrophages

Yolk sac macrophages first appear in the YS blood islands at E9 (albeit in small numbers) with a unique pattern of differentiation that bypasses the monocytic intermediate stage seen in adult macrophages (50, 52). YS-derived primitive macrophages spread into the embryo proper through the blood as soon as the circulatory system is fully established (from E8.5 to E10) (56), and migrate to various tissues, including the brain. Importantly, this occurs before the onset of fetal monocyte production by the FL, which starts around E11.5/E12.5 (92). These primitive macrophages retain the high proliferative potential observed in the YS as they colonize various tissues (52, 93–95). Primitive macrophages may contribute to many fundamental processes during mid and late embryogenesis, such as clearance of dead cells or tissue maturation. In this regard, the developmental process of interdigital cell death removal during the mouse footplate remodeling that occurs between E12.5 and E14.5 is of interest as the interdigit regions become heavily populated by macrophages and most of the dead cells were shown to be rapidly engulfed by macrophages (96). However, mouse models devoid of primitive macrophages such as the colony-stimulating factor 1 receptor (CSF-1R) KO (Florent Ginhoux, unpublished data,) and PU.1 KO (97) appears to exhibit a normal interdigit web tissue. Wood et al. observed that interdigit web tissue in PU.1 KO was only slightly retarded, suggesting that other cell type such as neighboring mesenchymal cells were compensating (97). In addition, we recently showed that depletion of primitive macrophages and hence of embryonic microglia, affected the progression of dopaminergic axons in the forebrain and the laminar positioning of subsets of neocortical interneurons, likely through phagocytic mechanisms (98).

Schulz et al. highlighted further differences between primitive and definitive hematopoiesis, showing that the latter relies on the transcription factor Myb, while YS-derived macrophages are Myb-independent, and are instead dependent on PU.1 (12). This again reinforces the view that YS-derived macrophages constitute an independent lineage, distinct from the progeny of definitive HSCs. Schulz et al. exploited the differential dependence of primitive versus definitive hematopoiesis on the transcription factor c-Myb and reported that E16.5 tissue macrophage populations were not affected by the loss of c-Myb. Using a CSF-1R-iCre fate-mapping model of YS macrophages, they also reported the persistence of YS macrophages progeny in adult tissue-resident macrophage populations (lung, liver, and pancreas, as well as in the brain and skin), although the level of labeling was minimal (below 3–5%) and decreased with time. The authors concluded that tissue-resident macrophages were therefore derived from a c-Myb-independent lineage via YS macrophages (12), data supporting the initial report showing that microglia arise from YS macrophages (9). Embryonic origin of macrophages was further supported by the work of Yona et al. and Hashimoto et al. showing that adult monocytes do not substantially contribute to tissue macrophages under steady-state conditions (13, 33). Furthermore, Yona et al. suggested the existence of a CX3CR1^+^ precursor for some of the monocyte-independent macrophages, although the exact nature of this precursor was not elucidated. In fact, both YS macrophages and fetal monocytes express CX3CR1 (9, 11, 15) and could therefore correspond to the unidentified precursors suggested by Yona et al. However, using a CSF-1R-iCre fate-mapping model, also used by Schulz et al. (12), another study noted that the YS macrophage contribution in the brain, the adult liver, and the heart was maintained although at a minimal level that decreased with time (99). Interestingly, the level of labeling was always higher in microglia than that in the liver or the heart, suggesting that the level of YS macrophage contribution may differ between tissues and that YS macrophages may be differently replaced over time by later waves of progenitors, which follow tissue-distinct kinetics (discussed below). Our own report using the Runx1-iCre fate-mapping model (86) indicated that only microglia, specialized macrophages of the central nervous system, were derived solely from primitive macrophages while all other tissue macrophages derived from definitive hematopoiesis (9).

To understand whether YS macrophages might be the sole progenitors of every other adult macrophages, we asked what impact their *in utero* depletion would have on the subsequent generation of fetal tissue macrophages. CSF-1R is expressed on YS macrophages and fetal monocytes, but only the development of the former is actively dependent on CSF-1R (9, 11). Thus, we attempted to deplete YS macrophages by transiently inhibiting the CSF-1R signaling pathway using a blocking anti-CSF-1R antibody, as recently described (98). Importantly, after complete depletion of primitive YS macrophages in E10.5 embryos and thus of most macrophages in treated embryos at E14.5, tissue macrophages (including microglia) were able to repopulate to normal levels before birth. These data suggest that YS macrophages are dispensable for the generation of tissue-resident macrophages in the embryo, and that another CSF-1R-independent embryonic precursor can functionally replace YS macrophages during development (15, 98). Using a combination of both the CSF-1R-iCre and the Runx1-iCre fate-mapping models, we noted that although YS macrophages infiltrate all tissues (including lung, liver, kidney, skin, gut, heart, pancreas, and stomach) until E13.5, a second wave of precursors, with a monocytic morphology and phenotype, supersedes them after E14.5 with the exception of the brain where YS macrophages are maintained until adulthood (15). A fuller understanding of this process may help to resolve some of the earlier discrepancies regarding the contribution of YS macrophages.

### Fetal monocytes

Fetal monocytes were described by Naito et al. (92). Focusing their study on liver Kupffer cells (the resident macrophages of the liver) during embryonic development, they exploited the endogenous peroxidase activity of monocytes and pro-monocytes granules described earlier by van Furth et al. (18, 92). Naito et al. observed the transient appearance of peroxidase activity, a signature for monocyte and pro-monocyte granule activity, during the *in vitro* generation of macrophages from a preparation of FL-dissociated cells (92). In the YS and at early stages of FL development, no peroxidase activity was observed, suggesting that primitive macrophages first seed the FL. At a later stage, the peroxidase activity increased, suggesting the presence of monocytic intermediates. *In vitro* clonal expansion assays confirmed the existence of two types of colonies, those containing fetal monocytes and those devoid of them. This provided early evidence for the existence of two distinct developmental pathways leading to the generation of Kupffer cells, although at this stage, direct differentiation of fetal monocytes into macrophages *in vivo* had not been demonstrated (100).

To investigate the developmental event leading to the emergence of tissue-specific macrophages, we initially focused on the LC, the specialized myeloid population of the epidermis. While YS macrophages seed the embryonic skin before E13.5, we discovered that the major fraction of adult LCs is in fact derived from fetal monocytes that are generated in the FL from E12.5 and are then recruited into fetal skin at E14.5 (11). These cells share a similar phenotype to their adult counterparts; however, they are generated independently of CSF-1R expression (9, 11). They possess high proliferative potential, and, in contrast to their adult counterparts, express few genes related to pathogen recognition and immune activation (15). Further studies should clarify whether such differences reflect monocyte immaturity imposed by a sterile fetal environment, or rather dedicated functional specializations that have yet to be unraveled. *In utero* adoptive transfers combined with fate-mapping studies unequivocally confirmed *in situ* differentiation of fetal monocytes into adult LCs (11). Fetal monocytes were then demonstrated to be the precursor of adult macrophages in lung alveoli by intranasal injection (10, 101). Fetal monocytes were also shown to be involved in the generation of adult macrophages of the heart (99). In fact, fetal monocytes become the major leukocyte within the blood circulation after E13.5, spreading to all tissues. This occurred independently of the CCL2/CCR2 axis (15), suggesting an alternative mechanism of exit from the FL and/or recruitment by fetal tissues. Moreover, we were able to fate-map, from before birth to adulthood, the local differentiation of fetal monocytes into resident macrophages, by taking advantage of the specific expression of S100a4 in fetal monocytes compared to YS macrophages (15). Only the brain remained free from fetal monocyte infiltration, possibly resulting from the isolation of the brain by the nascent blood–brain barrier as early as E13.5 (15, 102). Thus, these data now reveal that fetal monocytes are the major circulating embryonic precursor for all macrophages, with the exception of the brain. The absence of monocyte precursor contribution to the microglial pool could result from a lack of intrinsic potential or a lack of access to the developing brain due to the nascent blood–brain barrier. Interestingly, we observed a major influx of monocytes in the brain at E14.5 in our YS macrophage depletion model, and preliminary data using our fetal monocyte S100a4-Cre/WT fate-mapping model combined with *in utero* depletion of YS macrophages suggest that fetal monocytes are capable of giving rise to microglia under certain conditions (Hoeffel & Ginhoux, personal communication). Whether this atypical fetal monocyte infiltration reflects a compensatory mechanism to fulfill an empty niche in the brain or results from a disruption of the blood–brain barrier remains to be investigated.

### Adult monocytes

BM-derived circulating monocytes were considered the only precursors for all tissue-resident macrophages since the seminal work of van Furth et al. (17–18). Although this dogma was entirely revisited recently with the emergence of sophisticated fate-mapping tools as well as parabiotic models, the physiological contribution of circulating adult monocytes to the adult macrophage network remains valid at least in certain tissues. The continuous recruitment of circulating monocytes to the dermis has been shown to shape the adult dermal macrophage network (25). Although this study did not employ fate-mapping techniques, Tamoutounour’s data suggest the existence in the dermis of both a prenatal pool of macrophages and a second pool derived from adult blood monocytes. The authors argue that the dermis, in contrast to the epidermis, continues to recruit circulating monocytes in adulthood, most likely facilitated by its high level of vascularization. The macrophage network in the intestine follows a similar model. Data from Bain et al. suggest that embryonic macrophages do not persist in adulthood in the gut, and are replaced constantly by circulating adult monocytes (23), convincingly showing that adult monocytes are the source of intestine-resident macrophages. The role of commensal microbiota in this process is supported by the observation that the use of germ-free animals or treatment with broad-spectrum antibiotics results in a significant reduction in the recruitment of Ly6C^+^ monocytes to the colon (23). The macrophage network of the heart has also been shown to contain a component of YS macrophages and fetal monocyte-derived macrophages, both of which are maintained in adulthood (99). However, similar to the dermis and the gut, adult monocytes seem to replace embryonic macrophages progressively over time (24). The decreasing capacity for self-renewal of embryonic macrophages with age observed by Molawi et al. may explain the requirement for continuous recruitment of monocyte-derived macrophages to the heart in the absence of inflammation. It remains to be clarified whether this phenomenon occurs in other tissues as a result of aging. In agreement, proliferation of YS macrophages and fetal monocytes is very high during development (20–40% before E14.5) but decreases progressively to 10% few days after birth in most tissues and decreases to almost undetectable levels in adults (15). Interestingly, macrophage turnover seems different from one tissue to another. Following BrdU incorporation at steady state, almost no proliferation was observed in adult gut macrophages (23), while 2–5% was measured in adult heart macrophages (24). Macrophage proliferation activity can also be mobilized upon inflammation. For example, peritoneal macrophages can increase their proliferation rate from 1 to 9% in response to parasite infection or in response to IL-4 stimulation (35), while enhanced local proliferation of macrophages in atherosclerotic lesions sustain disease progression (103). The characterization of local signals regulating macrophage proliferation as well as the presence of specialized tissue niches that sustain macrophage survival, proliferation, or even “stemness” will be fundamental to better understand their tissue homeostasis.

The macrophage network of the lymphoid system seems to follow a similar pattern than in the gut and dermis. Although the lymph nodes (LN) start to develop very early in the embryo (104), they become functionally active only within the first week after birth recruiting and organizing B and T cell areas when follicles start to shape with connections to afferent lymphatics via the subcapsular sinus (105). Although macrophages are known to participate in lymphangiogenesis during development, notably by the production of VEGF (106, 107), the precise origin of the different LN macrophage populations remain poorly understood (108). The high level of foreign antigens passing through the LN during the lifespan, support the model of a constant replenishment of the local macrophage pool by circulating adult monocytes. However, the work of Jakubzick et al. suggests otherwise as tissue-patrolling monocytes at steady state seem to enter the LN without any sign of local differentiation to macrophages or dendritic cells (34). Further studies using fate-mapping systems should be addressed to clarify this point. Spleen macrophages are generated prenatally (13, 33). However, red pulp macrophages and marginal zone macrophages seem highly dependent, respectively, on the transcription factor SPI-C (109) and on the nuclear receptor LXR (110), also expressed by circulating monocytes and suggest again that embryonic-derived macrophages are replaced over time by adult monocytes-derived macrophages. The use of the S100a4-Cre fate-mapping model in our hands supports these observations and similar conclusions were obtained for BM and peritoneal macrophages (15). Although tissue microenvironment shapes certain macrophage functional specificities (111), through an ontogenic point of view, the composition of each tissue-resident macrophage pool evolves throughout life and the respective origins of each macrophage population may account for some of their key functions and cellular behaviors in a given tissue. Hence, a new challenge is to understand if an embryonic or adult origin matters for the function and the activation states of tissue-resident macrophages.

## Origin and Development of YS Macrophages and Fetal Monocytes

### Origin of YS macrophages

Bertrand et al., in line with the seminal work of Palis (45), described two sequential myeloid waves within the early YS (42). Using an *in vitro* culture reporter system, Bertrand et al. observed a first wave of monopotent progenitors that gave rise only to macrophages, followed by a second wave that gave rise to a mix of granulocytes, monocytes, and macrophages. More recently, Kierdorf et al. revisited the work of Bertrand et al. exploiting organotypic embryonic brain slices to demonstrate that microglial cells derived from YS EMPs (112). Kierdorf et al. also showed that these EMPs did not express the transcription factor c-Myb, associating them with the progenitors reported by Schulz et al. (12), although a direct link with the generation of microglia *in vivo* in adulthood was not conclusively demonstrated. More recently, Perdiguero et al. used the CSF-1R-iCre fate-mapping model to show that YS macrophages are derived from CSF-1R^+^ EMPs (14). Hence, these two studies suggest that YS macrophages, and thus microglia, would originate from c-Myb-independent CSF-1R^+^ EMPs. Furthermore, Perdiguero et al. demonstrated that CSF-1R^+^ EMPs were able to seed the FL by E10.5, suggesting that these progenitors could later populate other tissue niches and produce YS-like macrophage later during development in others tissues. Nevertheless, these data do not explain the low percentage of labeled adult macrophages observed by Schulz et al. using the CSF-1R-iCre fate-mapping model (12). Later observations by Epelman et al. (99), and more recently by our group using the same fate-mapping model (15), indicated that the ability of CSF-1R^+^ EMP to reach the FL could explain the surprising maintenance of primitive macrophages until E16.5 in c-Myb null embryos, where primitive macrophages generated in the YS as well as in the FL would be able to fulfill the empty niche left by the absence of c-Myb-dependent myeloid cells, that include fetal monocytes. However, this may not reflect the physiological situation and may instead result from a compensatory mechanism to ensure the presence of macrophages in all tissues in the absence of c-Myb activity and fetal monocytes. Using the same CSF-1R-iCre fate-mapping model (15), we were able to follow the maintenance of microglia in the brain by self-renewal from E10.5 until adulthood, linking them with CSF-1R^+^ EMPs and confirming the previous observations of Perdiguero et al. (14). However, for all other macrophage populations, the reduction of fate-mapping reporter labeling after E13.5 confirmed the progressive replacement of YS macrophages by another unlabeled precursor arising from a different hematopoietic wave.

We previously showed that Runx1^+^ YS progenitors that emerged at E7.5 give rise to YS macrophages and microglia (9, 11). Using both the Runx1-iCre and the CSF-1R-iCre fate-mapping models, we showed that these E7.5 Runx1^+^ YS progenitors were in fact the same CSF-1R^+^ EMPs described by Perdiguero et al. and Kierdorf et al., which contributed to the generation of YS macrophages and, to a lesser extent, those seeding the FL (14, 15, 112). However, we also observed their disappearance from the FL after E11.5 indicative of a rapid local consumption/differentiation rather than long-term maintenance. Our results also suggest that these early CSF-1R^+^ EMPs are able to contribute to a short-term maintenance of macrophages in the FL (Figure 2), but do not contribute to other tissue macrophages as evidenced by their rapid disappearance from the blood circulation after E14.5 (15). This transient population in the FL may be due to a local immediate requirement for macrophages, at least during the onset of FL hematopoiesis, to perform efficient enucleation of primitive erythrocytes passing through the FL sinusoids (100, 113). Combining historical evidences showing their direct lineage connection with the emergence of YS macrophages and recent findings showing their independence with c-Myb activity, we propose that CSF-1R^+^ EMPs should be designated as primitive EMPs.

### Origin of fetal monocytes

Because adult monocytes are derived from HSCs in the BM, it would be reasonable to assume that embryonic HSCs might also give rise to fetal monocytes in the developing liver. In agreement with this hypothesis, we have identified a population in the FL similar to adult MDPs that have the potential to generate fetal cMoPs and monocytes following *in vitro* culture (15). Exploiting the Flt3-Cre tomato fate-mapping model (83), we then followed the progeny of embryonic HSCs. However, the poor labeling observed between E14.5 and E17.5 in FL monocytes and macrophages contrasted with the strong labeling of FL MDPs, suggesting that HSCs had limited involvement in the generation of fetal monocytes (15). Nonetheless, the limited but significant labeling in fetal monocytes and macrophages at birth suggested an increasing derivation from fetal HSCs, assuming that fetal HSCs follow a similar Flt3-dependent differentiation pathway as adult HSCs. In parallel, gene array analysis highlighted a strong lymphoid signature within fetal MDPs (15), indicative of their derivation from the recently described YS-derived LMPs (67). Thus, LMPs may be important for the generation of a small but significant proportion of fetal monocytes prior to the expansion of mature HSCs (Figure 4). Further investigations using more specific fate-mapping models will be necessary to elucidate the exact contribution of LMPs as well as the hematopoietic transition between the FL and the BM.

Importantly, we observed that fetal monocytes were not tagged with the CSF-1R-Cre model that label early CSF-1R^+^ EMPs, suggesting that fetal monopoiesis is not dependent on CSF-1R^+^ EMPs, consistent with our previous data (9, 11) and with our YS macrophage depletion results (15, 98). Furthermore, the Runx1-iCre fate-mapping model allowed us to identify two waves of EMPs that arise sequentially before LMPs in the YS. These included an early wave, arising at E7.5 that differentiates locally into YS macrophages; and a later wave tagged at E8.5, that migrates and seeds the FL following the establishment of the blood circulation before E9.0. Early EMPs tagged at E7.5 were therefore related to those described previously by Kierdorf and Perdiguero (14, 112). The late EMPs tagged at E8.5, however, expressed c-Myb, expanded more efficiently in the FL, and differentiated *in vivo* into fetal cMoPs, constituting the major component of the fetal monocyte population as well as the fetal monocyte-derived macrophage population (Figure 3), which was able to maintain itself in all tissues tested (15).

The existence of two distinct EMP waves is in agreement with Bertrand et al. who reported an early wave of macrophage progenitors restricted to the YS, and a second wave that was able to reach the FL to participate in definitive hematopoiesis (42). The differential expression of c-Myb between early and late EMPs is in agreement with previous reports indicating that primitive hematopoiesis can occur in the absence of c-Myb, especially for the generation of monopotent macrophage progenitors (114), whereas EMPs from definitive hematopoiesis express and are dependent on c-Myb activity (45, 62, 115).

Notably, a previous study showed that c-Myb ablation strongly compromises definitive hematopoiesis (116). Palis et al. observed that c-Myb is expressed prior to and during the early development of definitive erythrocyte progenitors (45). Thus, late EMPs and LMPs, as well as HSCs, express c-Myb (15, 45, 61, 62), suggesting that the entire fetal monopoiesis machinery is reliant on this transcription factor. In agreement, the CD11b^hi^F480^lo^ population, which in our hands contains fetal monocytes, was completely absent in the c-Myb-deficient embryo (12, 116). As a consequence, the contribution of c-Myb-dependent progenitors to tissue-resident macrophage populations could not be evaluated in c-Myb-deficient embryos, where c-Myb-independent YS macrophages maintain themselves as a compensatory mechanism due to the absence of c-Myb-dependent fetal monocytes that normally outcompete them. Because c-Myb expression is upregulated during the successive steps of fetal monopoiesis (15), the switch in EMP localization between the YS and the FL may indeed be orchestrated by c-Myb. As a consequence, most tissue-resident macrophages derived from fetal monocytes would therefore rely on c-Myb activity. Altogether we propose that c-Myb^+^ EMPs giving rise to the first circulating monocytes should be designated as definitive EMPs.

## Conclusion

Recent reports have drastically changed the view of the development of the MPS and shed light on the multiple layers that define fetal hematopoiesis. It is now evident that fetal monocytes form the major precursors of most adult tissue-resident macrophages, and further investigations are now necessary to clarify how they shape macrophage heterogeneity. Examining how tissues imprint specific fates in these circulating precursors will aid our understanding of the mechanisms that control the tissue-specific functions of macrophages in the steady state, and thus may uncover new therapeutic opportunities in diverse pathological settings such as metabolic diseases, fibrosis, and carcinogenesis.

## Conflict of Interest Statement

The authors declare that the research was conducted in the absence of any commercial or financial relationships that could be construed as a potential conflict of interest.

## Funding

This work was supported by the Singapore Immunology Network (SIgN) core grant.
